# Continued Access to Investigational Medicinal Products for Clinical Trial Participants—An Industry Approach

**DOI:** 10.1017/S0963180118000464

**Published:** 2019-01

**Authors:** ARIELLA KELMAN, ANNA KANG, BRIAN CRAWFORD

**Keywords:** continued access, corporate responsibility, post-trial access, research ethics, low and middle income countries

## Abstract

In the conduct of clinical trials for pharmaceutical research, access to investigational medicines following clinical trials is often necessary for the continued health and well-being of the trial participants; it is an ethical obligation under some circumstances, as outlined in the Declaration of Helsinki 2013 Article 34. This obligation becomes particularly important in lower-income countries, where access to medical care may be limited. Although there is agreement among global research and bioethics communities that continued access should be provided with prospectively defined parameters and procedures, the process is complex, as many responsible parties and complicated logistics are involved. Roche Pharmaceuticals developed and publicly posted the company’s policy regarding continued access to investigational medicines in 2013. This article provides insights on the policy, including the parameters that determine when continued access is and is not considered to be appropriate, along with an example from an active clinical development program. It also describes how multiple stakeholders, including those in academia, industry, government, and patient advocacy, have worked together to assess approaches to continued access. Continued access plans should be transparent and agreed to by research participants, investigators, and governments prior to the study and reassessed based on clinical trial evidence of safety and efficacy and availability of adequate treatments, along with relevant international laws and customs. Conducting responsible continued access programs requires close partnerships with investigators, health authorities, and third-party research partners.

## Introduction

The TRUST Project, funded by the European Commission under the HORIZON 2020 program, aims to foster adherence to high ethical standards and prevent double standards in research ethics, particularly when researchers from high-income countries (HICs) conduct research in lower- and middle-income countries (LMICs). The focus of TRUST is to improve “North–South research collaborations,” in which research leads or sponsors based in HICs (usually in the Northern Hemisphere) conduct research in low-income (usually Southern Hemisphere) regions.^1^ The scope of TRUST is broad, and includes clinical and other types of research involving academia, industry, and others. Some of the proposed goals of the project include developing tools for vulnerable populations to help improve global research governance structures.

In the specific example of clinical trials for pharmaceutical research, access to investigational medicines following clinical trials is often necessary for the continued health and well-being of the trial participants. It is also considered an ethical obligation under some circumstances, as outlined in the World Medical Association’s Declaration of Helsinki 2013 Article 34.^2^ This obligation becomes particularly important in lower-income countries, where access to medical care may be limited. Roche developed and publicly posted the company’s policy regarding continued access^3^ to investigational medicines in 2013. For this reason, and given the importance of the topic, representatives from Roche were invited to a meeting of the TRUST project in June 2017 in London, U.K., to discuss our approach and present an example based on clinical trials for a chronic disease. This paper represents a summary of that discussion.

The benefits and risks of new, innovative treatments for highly unmet medical needs are typically assessed in clinical trials involving eligible volunteer patient participants. Study sponsors, who are often pharmaceutical companies, search worldwide for optimal study sites to enable the research. Several considerations determine study site selection, including the epidemiology of the disease under study (e.g., is the disease more common in some regions?), and the availability of necessary infrastructure to conduct the trial in a manner that is both ethical and scientifically valid (e.g., incorporates content expertise of the investigator and institution, including necessary training, equipment, and experience with relevant medical care and research).

## Approach to Continued Access to Investigational Medicines at Roche

A clinical trial is not a treatment protocol or a replacement for medical care, but rather a scientific experiment designed to test a specific hypothesis. The outcomes are not known at the start, and it is important for patient participants to carefully assess the potential risks and benefits during the informed consent process before agreeing to trial participation. Participation in research is not synonymous with routine medical care. Nevertheless, the parties involved in conducting a clinical trial, including sponsors, investigators, treating physicians, and health care systems (including governments), acknowledge that patients with serious diseases, particularly chronic diseases, should have access to care following a clinical trial and that under certain circumstances this care should include the investigational agent, if successfully tested. Depriving a patient of an effective treatment that they received in a trial, when no other equivalent treatment exists or is available, is considered exploitative and unethical. In regions with limited access to health care there is an even higher risk of exploitation. It follows that the goals of continued access include minimization of exploitation of research participants, particularly in LMICs, and solidification of trust among investigators and participants.^4^

The Declaration of Helsinki, Paragraph 34, “Post-Trial Provisions,” states that

In advance of a clinical trial, sponsors, researchers, and host country government should make provisions for post-trial access for all participants who still need a product identified as beneficial in the trial. This information must also be disclosed to participants during the informed consent process.^5^

Guideline 6 (Caring for Participants’ Health Needs) of the relevant guidelines of the Council for International Organizations of Medical Sciences notes that “addressing participants’ health needs requires at least that researchers and sponsors make plans for . . . providing continued access to study interventions that have demonstrated significant benefit.”^6^

Although there is agreement among global research and bioethics communities that continued access should be made available with prospectively defined parameters and procedures, the process is complicated, as many responsible parties (e.g., sponsors, researchers, and host country governments) as well as complicated logistics are involved. Roche developed a policy on continued access to investigational medicines to ensure that continued access obligations to research participants are consistently and sustainably met based on evidence from clinical trials and in alignment with the Declaration of Helsinki. The policy was codeveloped by Roche researchers and clinical trial professionals together with the Roche Scientific Ethics Advisory Group. The Scientific Ethics Advisory Group is composed of international experts invited by Roche from the fields of genetics, bioethics, law, and science policy, as well as patient advocates.^7^ The policy is shown here (**Box**).^8^

**Roche Global Policy on Continued Access to Investigational Medicinal Product**

**Executive Summary**

Roche is committed to a high standard of quality and ethical conduct in all aspects of conducting clinical trials. As part of this commitment and in accordance with the Declaration of Helsinki, Roche offers patients who participate in Roche-sponsored clinical trials continued access to the investigational medicinal product that they received after trial completion, when appropriate, as described below.

**Global Policy**

The purpose of this Global Policy is to describe the principles that govern when a patient who participates in a Roche-sponsored clinical trial of an investigational medicinal product shall have continued access to that product after completion of the clinical trial, free of charge, if:

1) The patient has a life threatening or severe medical condition and his/her wellbeing requires continued administration of the investigational medicinal product;
2) There are no appropriate alternative treatments available to the patient; and
3) The patient and his/her doctor comply with and satisfy any legal or regulatory requirements applicable to them.

**Exceptions**

Roche will not provide continued access to investigational medicinal product as described above if:

1) The investigational medicinal product is commercially marketed in the patient’s country and is reasonably accessible to the patient (e.g., is covered by the patient’s insurance or wouldn’t otherwise create a financial hardship for the patient);
2) Roche has discontinued development of the investigational medicinal product or data suggest that the investigational medicinal product is not effective for the relevant indication;
3) Roche has reasonable safety concerns regarding the investigational medicinal product for the relevant indication; or
4) Provision of investigational medicinal product would not be permitted under the laws and regulations of the patient’s country.

In addition to this high-level policy, Roche has developed an internal standard operating procedure to ensure that training for employees on the principles and operational considerations for continued access is performed. For example, all Roche-sponsored protocols and informed consent forms should include a general plan for continued access. When continued access is not appropriate, per the policy, this should also be made clear in the protocol and informed consent documentation, and agreed to by all parties.

Several mechanisms are utilized for providing continued access to investigational medicine to research participants who have completed a clinical trial. These include:

1) Open-label^9^ clinical trial (includes open-label extension studies and rollover studies): a continuation of a trial or a new trial for patients who have completed a clinical trial. In these types of trials, all patients receive investigational medicines. For rollover studies, patients are grouped from several clinical trials into one trial or from different cohorts within a single clinical trial. This is done in situations when data collected from such trials continues to be useful for understanding safety or efficacy of the medicine being studied.
2) Post-trial access programs: implemented when further research data on efficacy is not needed. In this case, individual patients who completed a trial can be provided with investigational medicines outside of a clinical trial. Data on safety events continue to be collected per a safety reporting agreement with the treating physician.
3) Patient support programs: used in some countries when the medicine is approved and available in the country’s health care system for the same indication but might not be accessible to some patients (e.g., because of lack of affordability or health plan coverage).

## Example of Multistakeholder Collaboration: The Multi-Regional Clinical Trials Center Post-Trial Responsibilities Working Group

**Figure 1.**
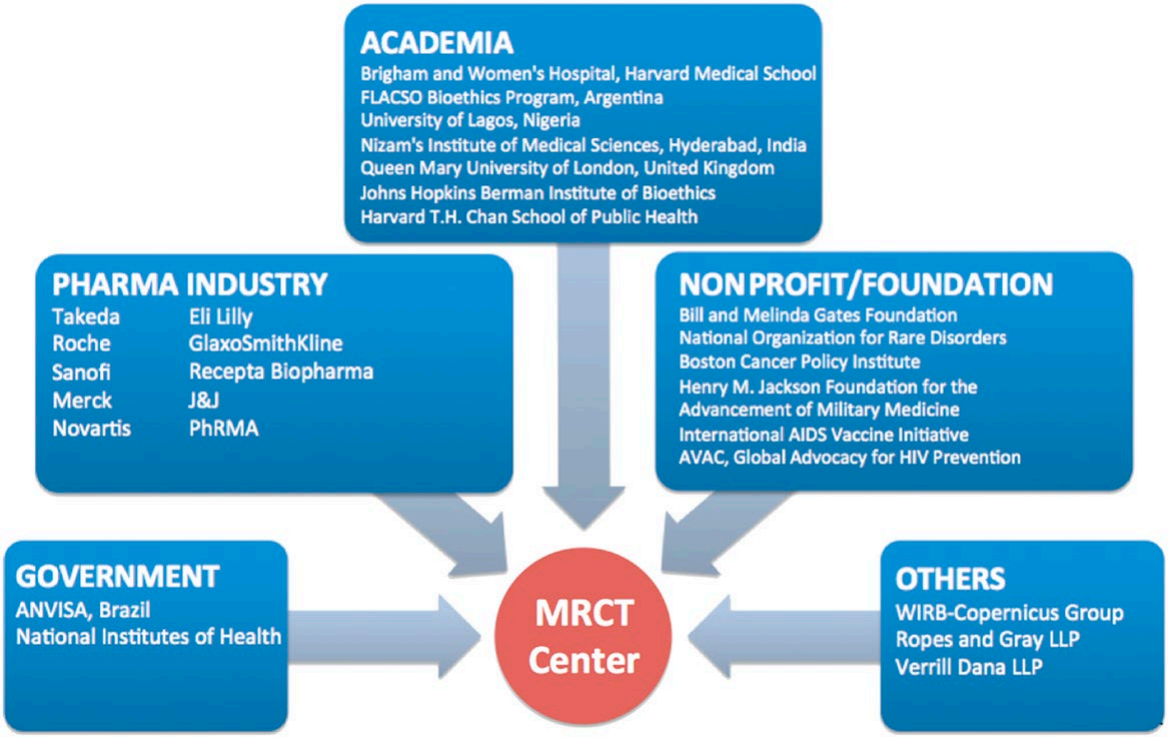
Continued Access Working Group

Given the medical, scientific, ethical, legal, and logistical complexities that are often involved in post-trial access decision making and implementation, particularly in large international trials, the Multi-Regional Clinical Trials Center of Brigham and Women’s Hospital and Harvard (MRCT Center), assembled a multistakeholder group during 2015-2017 to address these issues. The working group included members from academia, industry, governments, nonprofit foundations, patient advocacy groups, and the legal profession (**Figure 1**). The group has developed a Post-Trial Responsibilities Guidance Document to present an ethical framework for, and inform implementation of, post-trial access.^10^

The MRCT Center recommends that sponsors, investigators, governments, and healthcare systems collaborate before, during, and after a trial to perform pretrial planning, identify available alternatives to the investigational product, determine the risk-benefit profile for individuals and the study population (often after the end of a trial when trial data is available as to whether or not the investigational medicine is effective with an acceptable safety profile) and transition participants from the investigational product to routine medical care when appropriate. Post-trial access plans, to the extent that they can be known in advance of trial analysis, should be made transparent in the protocol and during informed consent processes, as required by the Declaration of Helsinki, see above. Responsibilities for post-trial access shifts over time from the sponsor to the local health care system, and plans need to be made to ensure that adequate resources are in place and research participants experience a smooth transition.

## Roche Clinical Trial Participation by Country Income Level

**Figure 2.**
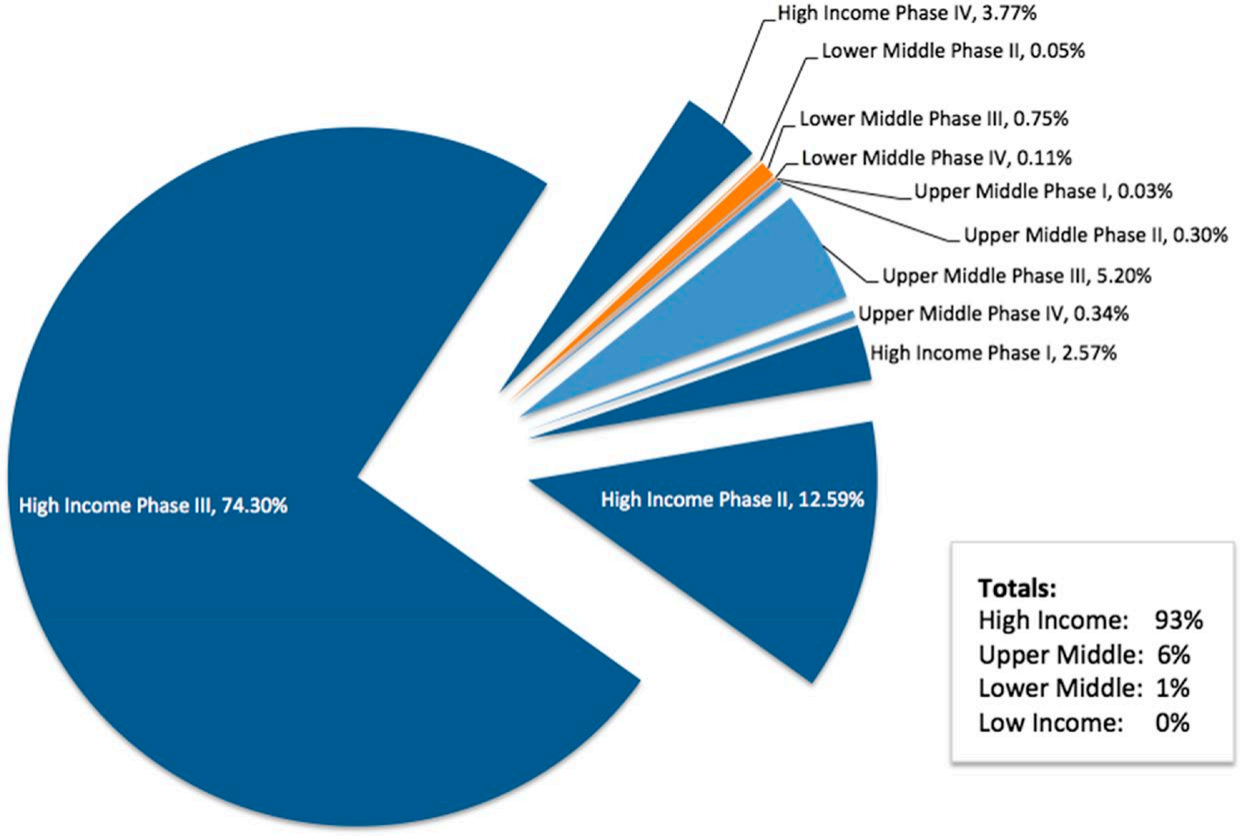
Roche Clinical Trial Participation by Country Income Level

The focus of the TRUST project is related to research in LMICs. In preparation for engagement with TRUST, we performed an analysis of the countries in which Roche conducts interventional clinical trials based on World Bank definitions of income.^11^ The analysis was performed in April 2017 with data obtained from the Roche Business Information Warehouse, a data mart that includes information from the clinical trial management system (**Figure 2**).

At the time of the analysis, approximately 1.5 million participants were enrolled in ongoing Roche-sponsored clinical trials worldwide, with more than 300,000 participants enrolled in more than 1,500 trials during 2016 alone. The Roche therapeutic pipeline is focused on serious medical conditions with significant unmet needs, including oncology, serious chronic inflammatory autoimmune diseases, and rare serious diseases. Many, but not all, of our investigational medicines are genetically engineered biologic therapies that require careful handling and storage under specific temperature and other conditions (e.g., cold supply chain). Many of our clinical trials also require shipping and handling of genetic material, biomarkers, and diagnostic test materials. These factors tend to require specialized facilities, including tertiary academic centers, for patient care and study conduct. This is a condition that is more difficult to meet for some LMICs, which do not have specific infrastructure for medical care and research.

We also aim to ensure that all study sites have appropriate oversight to ensure they can meet the high level of ethical conduct that Roche requires. Thus, the majority of Roche interventional clinical trials are conducted in HICs. In 2017, 93% of Roche-sponsored trial participation was in HICs, with 6% in upper-middle-income countries, and 1% in LMICs. Earlier-phase trials, when less is known about the benefits and risks of the investigational medicine, are typically performed in HICs.

Roche adheres to the same international regulations and applies the same high standards of ethical conduct and scientific integrity at all study sites across geographic regions. Furthermore, Roche intends to seek marketing authorization in all countries where Roche-sponsored clinical trials are conducted for the candidate therapy.^12^

## The Scope of the Continued Access Policy

The Roche Global Policy on Continued Access to Investigational Medicinal Products defines a particular scope (see Box 1). For instance, if the investigational medicinal product is commercially marketed in the patient’s country and is reasonably accessible to the patient (e.g., is covered by the patient’s insurance or wouldn’t otherwise create a financial hardship for the patient), Roche will not provide it after the trial ends. Once a medicine is approved for use by a country government, it is typically no longer investigational and may be available through the health care system. In these cases, it is most appropriate that individual patients receive the medicine through the usual local health care channels rather than directly from Roche. However, it is acknowledged that even after approval by a health authority, medicines may not be accessible to many research participants for some time, depending on the health care coverage plans of country governments and private payers. Roche assesses financial hardship or inaccessibility at the country level from which potential trial participants would be engaged; the assessment is ultimately entrusted to consultation between the local treating physician and the local Roche country affiliate clinical trial professionals.

The policy describes two other examples of situations in which continued access to investigational products would be inappropriate. This is the case if there are reasonable safety concerns regarding the investigational medicinal product for the relevant indication, and when the provision of investigational medicinal product would not be permitted under the laws and regulations of the patient’s country. It would not be appropriate to continue access in most situations when an investigational medicine has been shown to be ineffective, has demonstrated unacceptable safety risks in clinical trials, or manufacturing of the medicine has been discontinued. In these cases, the investigator and local health care system do have obligations to transition the research participant to appropriate standards of care.

At the same time, the Roche policy has three conditions where continued access would be appropriate. To implement the policy, it is necessary to clarify who *may* and who *may not* be eligible for continued access considerations. The policy enables post-trial access, with exceptions, according to three fundamental requirements (see Box 1):

1) The patient has a life-threatening or severe medical condition and his/her well-being requires continued administration of the investigational medicinal product;
2) There are no appropriate alternative treatments available to the patient; and
3) The patient and his/her doctor comply with and satisfy any legal or regulatory requirements applicable to them.

The first two requirements will be discussed here. The third is a straightforward requirement related to conduct within applicable laws and regulations. Determining whether a disease is a “life-threatening or severe medical condition” requires medical judgment. For practical purposes, this is taken to be consistent with definitions used by the U.S. Food and Drug Administration for “life-threatening or serious diseases,” where “life-threatening” is defined as “diseases or conditions where the likelihood of death is high unless the course of the disease is interrupted”; “the seriousness of a disease is a matter of judgment, and is based generally on such factors as survival, day-to-day functioning, and the likelihood that the disease, if left untreated, will progress from a less severe condition to a more serious one.”^13^

In addition, the fact that “there are no appropriate alternative treatments available to the patient” is regarded as an essential criterion for unmet medical need. An “appropriate alternative treatment” is an accepted regimen that is used to treat or control a medical condition. The availability of alternative treatments, including costs and payment structure (e.g., insurance/health plans), administration and monitoring infrastructure, and support by local health authorities, must be considered. A key consideration in determining the availability of an appropriate alternative is to ensure that research participants are not worse off after the study than they were during the study.

## Implementing the Continued Access Policy

The determination of whether post-trial access will be required for participants is initiated early in trial planning based on the conditions, study objectives and endpoints, and availability of alternative treatments. The post-trial access policies, mechanisms of access, and the restrictions of possible participating countries are reviewed, including the anticipated scope and duration of post-trial access. Roche medical scientists and clinical trial management professionals are responsible for evaluating the factors outlined in the policy. For example, the evaluation of whether appropriate alternative treatments exist at potential study sites is conducted in consultation with local medical specialists treating patients with the condition. Roche is less likely to initiate a clinical trial in countries or regions where basic health care services cannot be continued after trial completion. As described in the Declaration of Helsinki (see above), plans for and approaches to continued access should be outlined in the trial protocol and during informed consent processes.

The implementation of post-trial access requires clarification of important details throughout the process. For example, the role of the treating physician needs to be clear given that research physicians may not be available after the close of the trial. This means that the treating physician would have to provide ongoing care following a trial. As such, research participants may also receive care in a health care facility other than the one used in the clinical trial. Roche provides guidance to local affiliates in different countries to help them determine whether a treating physician and/or facility is qualified and adequate to administer and manage the investigational product and willing to comply with the applicable global and local regulatory requirements.

## The Example of Etrolizumab for Ulcerative Colitis Clinical Trials

The etrolizumab clinical trial program employs a typical approach to continued access for a serious chronic condition, ulcerative colitis. Etrolizumab is an IgG1 humanized monoclonal antibody targeting α4β7 and hence inhibiting both trafficking into and retention in gut mucosal tissues through α4β7 and αEβ7, respectively.^14^ Phase 3 clinical trials of etrolizumab for the treatment of ulcerative colitis are ongoing and plan to enroll up to 2,600 participants in 44 countries (68% HICs, 25% upper-middle income, and 7% LMICs, according to World Bank definitions).^15^ Following the primary analysis endpoints, participants enrolled in etrolizumab phase 2 or 3 trials can enter a 2-part open-label extension and safety monitoring trial (ClinicalTrials.gov number NCT02118584). Patients may receive open-label etrolizumab for up to 7 years after the first participant is enrolled or the study is terminated, as part of the open-label extension program.^16^ Following the end of the open-label extension, research participants who continue to require etrolizumab and meet criteria according to Roche’s continued access policy can continue to receive it outside the protocol on an individual basis. To ensure that appropriate trial evidence is considered, conditions for further continued access will be reassessed after the results of the trials are available. Trial results are typically assessed by physician scientists and statisticians from the sponsor company, physician investigators, and others (including trial steering committees or data monitoring boards consisting of academic experts), as well as health authority reviewers.

## Future Directions

Strong partnerships have been forged across stakeholder groups in the effort to galvanize equitable and ethical research practices worldwide and have gathered significant momentum in recent years. Multistakeholder organizations and projects such as the MRCT Center and the TRUST Project are relatively new but have made far-reaching strides in clarifying and solidifying paths to improved research conduct. Current and future challenges in continued access to investigational medicinal products will remain connected with the structural health system disparities seen in lower- vs higher-income countries. Continued access plans should be transparent and agreed to by research participants, investigators, and governments prior to the study and reassessed based on clinical trial evidence of the safety, efficacy, and availability of adequate treatments, along with relevant international laws and customs. Conducting responsible continued access programs requires close partnerships with investigators, health authorities, and third-party research partners. Logistics and decision making can be complex in a global environment, and collaboration with academic, government, patient advocacy, and other stakeholders is essential for maintaining trust and ethical research conduct.

## Notes

1. trust-project.eu. *TRUST Equitable Research Partnerships*; c 2015-2017 [cited 2017 July]; available at http://trust-project.eu (last accessed 28 Sept 2018).

2. World Medical Association. World Medical Association Declaration of Helsinki: Ethical principles for medical research involving human subjects. JAMA 2013;310(20):2191–4.2414171410.1001/jama.2013.281053

3. The terms *post-trial* and *poststudy* access are most often used in the academic literature to discuss whether research participants have a right to successfully tested medicines. We are using the term continued access in line with the latest Council for International Organizations of Medical Sciences ethics guidelines, see note 6.

4. SchroederD. Post-trial obligations. RECIIS: Electronic Journal of Communication, Information & Innovation in Health*.* 2008;1:63–73.

5. See note 2, World Medical Association 2013:2191–4.

6. Council for International Organizations of Medical Sciences. International Ethical Guidelines for Health-Related Research Involving Humans; 2016; available at https://cioms.ch/wp-content/uploads/2017/01/WEB-CIOMS-EthicalGuidelines.pdf (last accessed 12 Sep 2017).

7. Roche.com. *Resolving Ethical Conflicts: Scientific Ethics Advisory Group* [cited 2017 Aug 2]; available at https://www.roche.com/research_and_development/who_we_are_how_we_work/ethics_in_rd/ethical_conflicts.htm (last accessed 28 Sept 2018).

8. Roche.com. *Global Policy on Continued Access to Investigational Medicinal Product*; 2013 [cited 2017 Oct 10]; available at http://www.roche.com/dam/jcr:54941039-30fe-4b4c-86c0-5c453ef72776/en/policy_continued_access_to_investigational_medicines.pdf (last accessed 28 Sept 2018).

9. In an open-label clinical trial, both the researcher and the research participant know which medicine is being administered.

10. Multi-Regional Clinical Trials Center (MRCT) of Brigham and Women’s Hospital and Harvard. *MRCT Center Post-Trial Responsibilities Framework: Continued Access to Investigational Medicines. I. Guidance Document*; available at http://mrctcenter.org/wp-content/uploads/2017/06/2017-06-28-Post-Trial-Responsibilities-Guidance-Document-Version-1.1.pdf (last accessed 13 Aug 2017).

11. The World Bank. *World Bank Country and Lending Groups*; available at https://datahelpdesk.worldbank.org/knowledgebase/articles/906519-world-bank-country-and-lending-groups (last accessed 17 Aug 2017).

12. Roche Position on Clinical Research; available at http://www.roche.com/dam/jcr:d85a877d-c806-4fd7-9d82398d9ba4f70e/en/roche_position_on_clinical_research.pdf (last accessed 26 Jul 2017).

13. U.S. Department of Health and Human Services Food and Drug Administration Center for Drug Evaluation and Research. Information program on clinical trials for serious or life-threatening diseases and conditions. *Center for Biologics Evaluation and Research*; March 2002; available at https://www.fda.gov/downloads/regulatoryinformation/guidances/ucm126838.pdf (last accessed 28 Sept 2018).

14. UngarB, KopylovU. Advances in the development of new biologics in inflammatory bowel disease. Annals of Gastroenterology 2016;29(3):243–82736602410.20524/aog.2016.0027PMC4923809

15. See note 11, The World Bank 2017.

16. *Study for Participants with Ulcerative Colitis Previously Enrolled in Etrolizumab Phase II/III Studies* [cited 2017 Jul 26]; available at https://clinicaltrials.gov/ct2/show/NCT02118584?term=NCT02118584&rank=1 (last accessed 28 Sept 2018).

